# Sequential versus massively parallel strategies for molecular characterization of non-small cell lung cancer samples obtained by endobronchial ultrasound-guided transbronchial needle aspiration

**DOI:** 10.36416/1806-3756/e20250039

**Published:** 2025-07-31

**Authors:** Luís Vaz Rodrigues, Marta Viegas, Ana Filipa Ladeirinha, Ana Alarcão, Luis Taborda-Barata, Rosa Cordovilla, Vitor Sousa

**Affiliations:** 1. Departamento de Pneumologia, Instituto Português de Oncologia de Coimbra Francisco Gentil, Coimbra, Portugal.; 2. Faculdade de Ciências da Saúde, Universidade de Beira Interior, Covilhã, Portugal.; 3. Departamento de Anatomia Patológica, Laboratório de Patologia Molecular, Instituto Português de Oncologia de Coimbra Francisco Gentil, Coimbra, Portugal.; 4. Instituto de Anatomia Patológica e Patologia Molecular, Faculdade de Medicina, Universidade de Coimbra, Coimbra, Portugal.; 5. Centro de Investigação em Meio Ambiente, Genética e Oncobiologia, Faculdade de Medicina, Universidade de Coimbra, Coimbra, Portugal.; 6. RISE-Health, Faculdade de Ciências da Saúde, Universidade de Beira Interior, Covilhã, Portugal.; 7. CICS-UBI - Centro de Investigação em Ciências da Saúde e UBIAir - Centro Clínico e Experimental do Pulmão, Universidade de Beira Interior, Campus da Faculdade de Medicina, Covilhã, Portugal.; 8. Departamento de Pneumologia, Hospital Universitário de Salamanca, Complexo Assistencial Universitário de Salamanca, Salamanca, Espanha.; 9. IBSAL - Instituto de Investigação Biomédica de Salamanca, Complexo Assistencial Universitário de Salamanca, Salamanca, Espanha.; 10. Departamento de Anatomia Patológica, Unidade Local de Saúde de Coimbra, Coimbra, Portugal.

**Keywords:** Non-Small Cell Lung Cancer, Endobronchial Ultrasound-Guided Transbronchial Needle Aspiration, Sequential Molecular Profiling, Massively Parallel Next-Generation Sequencing, Actionable Mutations, Personalized Therapy

## Abstract

**Objectives:**

The advent of massively parallel next-generation sequencing (MP-NGS) offers potential advantages over sequential molecular profiling (SMP) in the management of non-small cell lung cancer (NSCLC). This study compares the two methodologies using samples obtained through endobronchial ultrasound-guided transbronchial needle aspiration (EBUS-TBNA), focusing on actionable mutation detection, turnaround time (TAT), and clinical outcomes.

**Methods:**

A retrospective analysis was conducted on NSCLC patients who underwent EBUS-TBNA and molecular characterization between January 2020 and December 2023. SMP and MP-NGS were compared in terms of actionable mutation detection rates, TAT, and impact on overall survival (OS).

**Results:**

Among 106 patients, MP-NGS demonstrated a significantly higher detection rate of actionable mutations compared to SMP (40.9% vs. 22.2%, p=0.042). The median TAT was slightly shorter with SMP than with externally outsourced MP-NGS (17 days vs. 23 days, p=0.076). Patients diagnosed via MP-NGS were more frequently allocated to targeted therapies (44.26% vs. 22.2%, p=0.038), which may have positively influenced overall survival (672 days vs. 138 days, p=0.053).

**Conclusion:**

MP-NGS provided superior diagnostic and clinical advantages over SMP in NSCLC, supporting its adoption as a standard diagnostic approach to enhance personalized therapy and improve patient outcomes.

## INTRODUCTION

Lung cancer (LC) currently ranks first in both incidence and mortality among all types of cancer worldwide,^1^ and is closely associated with tobacco epidemics.^2^ Non-small cell lung cancer (NSCLC), which accounts for over 85% of all LC cases,^3^ remains a diagnostic challenge, as it often presents asymptomatically until advanced stages, when surgery is no longer a viable option.^4^ At this point, understanding its subcellular characteristics becomes critical, as this can unveil therapeutic pathways with significantly improved efficacy and safety profiles.^5^ ^,^ ^6^ Endobronchial ultrasound-guided transbronchial needle aspiration (EBUS-TBNA) plays a key role in this context by enabling both the diagnosis and staging of NSCLC^7^ ^,^ ^8^ ^,^ ^9^ in a minimally invasive manner. The main challenge, however, lies in obtaining adequate samples to meet the requirements of both pathologists and molecular geneticists-fulfilling the threefold goal outlined in clinical guidelines: diagnosis, staging, and molecular characterization in a single procedure.^10^

While EBUS-TBNA is a safe and effective tool for diagnosis and staging,^8^ ^,^ ^9^ its reported yield for molecular profiling is variable, likely due to methodological heterogeneity.^11^ ^,^ ^12^ In a previous study, we found that 89.5% of samples obtained via EBUS-TBNA were satisfactory for *EGFR* testing, but only 81.3% were suitable for *ALK* assessment.^13^ In that investigation, the *EGFR* status was determined by real-time polymerase chain reaction (RT-PCR); if the results were negative, *ALK* gene rearrangements were subsequently assessed using fluorescence *in situ* hybridization (FISH). Despite encouraging results, a key limitation of sequential testing strategies became apparent: depletion of EBUS-TBNA-collected material between tests, particularly affecting downstream markers. With the growing number of clinically relevant molecular markers, a decline in sample utility can be expected when using sequential methods.^14^ Therefore, evaluating the potential of massively parallel (MP) molecular analysis-particularly through next-generation sequencing (NGS)-is increasingly relevant.^15^ Emerging data support the feasibility of MP-NGS in EBUS-TBNA samples, with reported yields ranging from 86.1% to 98%, depending on the gene panel size.^16^ ^,^ ^17^ However, some variability persists. 

Building on these findings, the present study aimed to compare sequential molecular profiling (SMP) with massively parallel next-generation sequencing (MP-NGS) in NSCLC samples obtained via EBUS-TBNA, evaluating feasibility, turnaround time (TAT), treatment strategies, and overall survival (OS). The objective was to clarify the differences between these methods and determine which approach better enhances diagnostic accuracy, reduces TAT, and supports personalized treatment decisions. 

## METHODS

A cross-sectional cohort study was conducted including patients with stage IV NSCLC, as defined by the 8th edition of the TNM classification,^18^ diagnosed between January 2020 and December 2023 at the Francisco Gentil Portuguese Institute of Oncology of Coimbra (IPOC-FG). The cohort was retrospectively established by identifying eligible patients who underwent simultaneous EBUS-TBNA and molecular characterization of NSCLC during this period. Patients were divided into two groups based on the molecular profiling strategy adopted. Between January 2020 and December 2021, SMP was performed in-house, whereas from January 2022 onward, molecular characterization was conducted using outsourced MP-NGS. The two strategies were compared in terms of sample adequacy, mutation detection rates, actionable mutations, and TAT. Additionally, treatment modalities and OS were evaluated.

All patients provided written informed consent, and the study was conducted as part of a PhD project approved by the Ethics Committee of the IPOC-FG (approval No. 23-2022). 

EBUS procedures were performed using a BF-UC180F bronchoscope (Olympus, Tokyo, Japan) under general anesthesia, with airway secured via a laryngeal mask. TBNA was carried out using 21G needles (ViziShot 2, Olympus, Tokyo, Japan). In accordance with institutional protocol, at least three needle passages were performed per lesion. Suction use was guided by lymph node vascular patterns^19^ and was withheld in cases of grade III/IV vascularity. 

Collected specimens were fixed in a 4% aqueous formaldehyde solution, centrifuged at 400×g for 15 min for cell block preparation from the pellet, and subsequently embedded in paraffin for histopathological examination.

SMP followed a stepwise strategy that was performed after immunohistochemistry, including PD-L1 assessment, as previously described by our group.^13^ Briefly, the workflow involved RT-PCR for *EGFR* mutation analysis using the Cobas^®^ EGFR Mutation Test v2 (Roche Diagnostics, Mannheim, Germany), a CE-IVD assay designed to detect 42 mutations across exons 18, 19, 20, and 21, including exon 19 deletions, L858R, T790M, G719X, S768I, and exon 20 insertions. Formalin-fixed paraffin-embedded (FFPE) tumor sections (5 µm) were reviewed by a pathologist, and manual microdissection was conducted for samples containing fewer than 10% tumor cells. DNA was extracted using the Cobas^®^ DNA Sample Preparation Kit (Roche Diagnostics, Mannheim, Germany), and amplification/detection was carried out on a Cobas^®^ z480 analyzer (Roche Diagnostics, Mannheim, Germany), according to the manufacturer’s instructions.

*ALK* and *ROS1* rearrangements were evaluated by FISH using 3-µm FFPE tissue sections. Samples with fewer than 100 viable tumor cells were excluded from the analysis. Following standard pretreatment, slides were incubated overnight with SPEC ALK (Z-2124, ZytoVision GmbH, Bremerhaven, Germany) or SPEC ROS1 (Z-2144, ZytoVision GmbH, Bremerhaven, Germany) dual-color break-apart probes. After post-hybridization washing, the slides were analyzed using a Leica DMI6000 B fluorescence microscope (Leica Microsystems GmbH, Wetzlar, Germany).

For MP-NGS, FFPE tumor blocks with ≥10% tumor content were selected. Genomic DNA/RNA was extracted using the MagMAX™ FFPE DNA/RNA Ultra Kit (Thermo Fisher Scientific, USA), and nucleic acids were quantified with a Qubit^®^ 3.0 fluorometer. Sequencing was performed on the Genexus platform (Thermo Fisher Scientific, USA) using the Oncomine Precision Assay GX, which detects mutations, copy number variations, and fusion variants across 50 cancer-related genes. The results were interpreted using the Oncomine Reporter to identify associated therapies.

To ensure comparability, actionable mutations were defined as *EGFR* mutations, as well as *ALK* and *ROS1* rearrangements, which were consistently tested in both approaches and align with international guidelines for targeted therapies.^6^

Data analysis was performed using IBM SPSS Statistics software (v27.0; IBM Corp., USA). Continuous variables were presented as medians and ranges, while categorical variables were reported as frequencies (n) and percentages (%). The Shapiro-Wilk test was used to assess the normality of continuous variables. Since the variables did not follow a normal distribution, non-parametric methods were employed. Pearson’s Chi-Square test was used to compare operational characteristics between the SMP and MP-NGS groups. TAT, defined as the interval from sample collection to final diagnosis (in days), was analyzed using the Mann-Whitney U test. Kaplan-Meier curves were used to estimate median survival times, and survival distributions were compared using the log-rank test. Multivariate Cox regression analysis was applied to identify independent predictors of survival. Collinearity diagnostics, including the variance inflation factor (VIF), were conducted to confirm the absence of significant multicollinearity. All statistical tests were two-sided, with p-values < 0.05 considered statistically significant.

## RESULTS

During the four-year study period, 106 patients with stage IV NSCLC underwent molecular testing on samples obtained via EBUS-TBNA. Of these, 45 were tested using SMP and 61 using MP-NGS.

Patients in both the SMP and MP-NGS groups were predominantly male (62.2% and 60.7%, respectively), with median ages of 67 and 69 years. Adenocarcinoma was the most common histological subtype (SMP: 91.1%; MP-NGS: 88.5%), and the majority of patients were classified as stage IVB (SMP: 68.9%; MP-NGS: 65.57%). No significant epidemiological or clinicopathological differences were observed between groups. Detailed results are presented in Table 1.

**Table 1:** Epidemiological and clinicopathological characteristics of the included patients.

Variable	SMP (n=45)	MP-NGS (n=61)	p-value
Sex, n (%) Male Female	28 (62.2) 17 (37.8)	37 (60.7) 24 (39.3)	0.870*
Age, median (min; max)	67 (38; 84)	69 (42; 86)	0.933^#^
Smoking history, n (%) Never smoker Former smoker Current smoker	11 (24.4) 15 (33.3) 19 (42.2)	21 (34.4) 19 (31.1) 21 (34.4)	0.519*
ECOG performance status 0 1 2 3	17 (37.8) 18 (40) 7 (15.5) 3 (6.7)	34 (55.7) 19 (31.1) 7 (11.5) 1 (1.6)	0.223*
Diagnostic procedure EBUS alone EBUS and EUS-b	28 (62.2) 17 (37.8)	40 (65.6) 21 (34.4)	0.722*
Type of sample Lymph node Tumor Left adrenal gland	32 (71.1) 11 (24.4) 2 (4.4)	39 (63.9) 20 (32.8) 2 (3.3)	0.635*
Histology, n (%) Adenocarcinoma Adenosquamous carcinoma† Combined adenocarcinoma and NE carcinoma† Squamous cell carcinoma	41 (91.1) 2 (4.4) 1 (2.2) 1 (2.2)	54 (88.5) 4 (6.6) 3 (4.9) 0	0.556*
Stage, n (%) IVA IVB	14 (31.1) 31 (68.9)	21(34.4) 40 (65.6)	0.720*

Legend: SMP, Sequential molecular profiling; MP-NGS, Massively parallel-Next generation sequencing; ECOG, Eastern Cooperative Oncology Group; EBUS, Endobronchial Ultrasound; EUS-b, Endoscopic Ultrasound (trans-esophageal) with the echobronchoscope; NE, neuroendocrine; *Pearson’s Chi-square test; ^#^Mann-Whitney U test. †In cases classified as adenosquamous carcinoma (n=6) and combined adenocarcinoma with neuroendocrine features (n=4), the diagnosis was suggested based on morphology and immunohistochemistry, performed on FFPE cell blocks obtained by EBUS-TBNA. In five of these cases (3 adenosquamous, 2 combined adenocarcinoma/NE carcinoma), the diagnosis was later confirmed using surgical biopsies from the primary tumor (n=2) or metastatic sites (pleura, n=1; subcutaneous tissue, n=2).

Regarding molecular profiling outcomes, adequate samples were obtained in the SMP group for *EGFR* analysis in 93.3% of cases, for *ALK* in 78.4%, and for *ROS1* in 75%, resulting in an overall success rate of 62.2%. Actionable mutations were identified in 22.2% (*EGFR*: 15.6%; *ALK*: 6.7%), while no *ROS1* rearrangements were detected.

**Figure 1:**
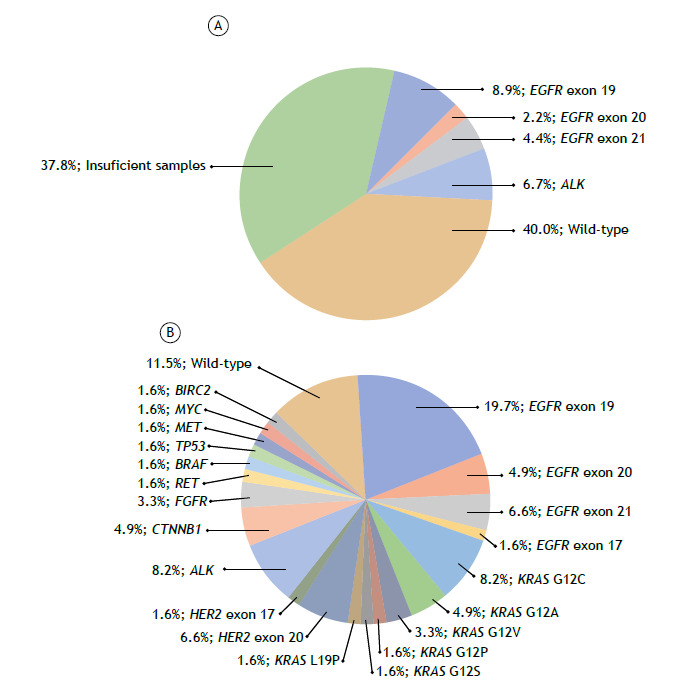
Pie charts illustrating the molecular profiling results in stage IV NSCLC samples using (A) sequential molecular profiling (SMP) and (B) massively parallel-next generation sequencing (MP-NGS). Legend: Aside from the data presented in the charts, 9 patients (14.7%) from the MP-NGS group exhibited complex molecular patterns: 1 harbored three mutations (*EGFR* exon 19, *CDKN2A*, and *PTEN*); 4 combined *EGFR* mutations with a second mutation (2 with *CTNNB1*; 1 with *TP53*; 1 with *PIK3CA*); 4 combined *KRAS* mutations with a second mutation (2 with *FGFR*; 1 with *TP53*; 1 with *BRAF*). These data highlight the superior discriminative power of MP-NGS, the absence of insufficient samples when using this method, and the reduced proportion of cases classified as wild-type.

In the MP-NGS group, all samples were adequate for molecular analysis. Mutations were detected in 88.5% of cases, with actionable mutations identified in 40.9% (*EGFR*: 32.8%; *ALK*: 8.2%). Similarly, no *ROS1* rearrangements were observed. However, additional relevant mutations were detected, including *HER2* (8.2%), *RET* (1.6%), and *BRAF* (1.6%). *KRAS* mutations were found in 21.3% of cases, with the G12C variant accounting for 8.2%. Details of the mutations are presented in Figure 1.

MP-NGS demonstrated significantly higher success in obtaining sufficient samples for molecular analysis (p=2 x 10^-5^) and enabled the identification of a significantly greater number of actionable mutations compared to SMP (p=0.042). A comparative summary of the operational characteristics of both methods is shown in Table 2.

**Table 2:** Comparison of molecular profiling techniques: SMP vs. MP-NGS.

Variable	SMP step 1 RT-PCR (EGFR )	SMP step 2 FISH (ALK )	SMP step 3 FISH (ROS1 )	SMP Overall results	MP-NGS Overall results	p-value
Patients tested, n (%)	45 (100)	37 (82.2)	24 (53.3)	45 (100)	61 (100)	NA
Adequate samples, n (%)	42 (93.3)	29 (78.4)	18 (75)	28^a^ (62.2)	61 (100)	2 x 10^-5^*
Samples with actionable^b^ mutations, n (%)	7 (15.6)	3 (6.7)	0	10 (22.2)	25 (41)	0.042*^$^
Time to positive result^c^, median (max; min)	8 (3; 34)	15 (9; 33)	NA	11 (3; 34)	24 (3; 57)	0.002^#^
Time to final molecular result^c^, median (max; min)	15 (9; 33)	17 (3; 58)	23 (3; 58)	17 (3; 58)	23 (3; 57)	0.076^#^

Legend: SMP, Sequential molecular profiling; RT-PCR, Real-time polymerase chain reaction; *EGRF*, Epidermal growth factor receptor; FISH, Fluorescence *in situ* hybridization; *ALK*, Anaplastic lymphoma kinase; *ROS1*, Proto-oncogene receptor tyrosine kinase; MP-NGS, Massively parallel-Next generation sequencing; NA, not applicable. ^a^Overall SMP: combines the positive results of *EGFR* (deemed complete, and that did not require further profiling) and the 18 additional cases where both *ALK* and *ROS1* could be tested, reflecting the sample sufficiency for all tests required to complete the molecular characterization of individual samples. ^b^Actionable Mutations: mutations assessed by all three diagnostic methods-*EGFR*, *ALK*, and *ROS1*-were considered actionable. ^c^Time to Result: the time, measured in days, from the completion of histopathological evaluation, including PD-L1 staining, to the final result of the molecular study. *Pearson’s Chi-square test; *^$^ Fisher’s Exact test; ^#^ Mann-Whitney U test.

The median TAT for positive results was significantly shorter with SMP than with MP-NGS (11 vs. 24 days; p=0.002). Although the overall TAT for SMP was also shorter than that of MP-NGS (17 vs. 23 days), this difference was not statistically significant (p=0.076). Detailed results for these measures are presented in Table 2. 

Considering therapeutic options, targeted therapy was administered to 44.3% of patients in the MP-NGS group, compared to 22.2% in the SMP group. Conversely, best supportive care was significantly less frequent in the MP-NGS group (13.1%) than in the SMP group (37.8%).

**Figure 2:**
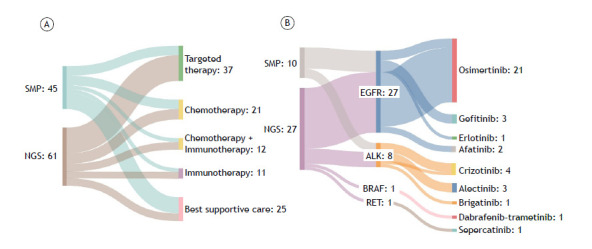
Relationship between molecular profiling strategies and first-line therapeutic choices with detailed targeted therapy selection. Legend: (A) Sankey diagram illustrating the distribution of first-line therapeutic strategies based on the molecular diagnostic method. The figure highlights a significant increase in the use of targeted therapies with MP-NGS compared to SMP (44.3% vs. 22.2%; p=0.026; Pearson’s Chi-square test) and a notable reduction in the use of best supportive care strategies with MP-NGS compared to SMP (13.1% vs. 37.8%; p=0.019, Pearson’s Chi-square test). (B) Sankey diagram detailing the targetable mutations identified by each method and their corresponding therapies. This panel underscores the superior discriminatory capacity of MP-NGS, which identified more actionable mutations and facilitated greater use of targeted therapies.

The differences between the SMP and MP-NGS methods were statistically significant regarding the increased use of targeted therapy (p=0.026) and the reduced utilization of best supportive care (p=0.019). Therapeutic allocation by profiling method (SMP vs. MP-NGS) and the relationship between detected actionable mutations and corresponding targeted therapies are detailed in Figure 2.

The Kaplan-Meier survival analysis revealed significant differences in OS based on the presence of actionable mutations (log-rank p=0.002) and first-line therapy (log-rank p<0.001). Patients with actionable mutations had a median OS of 1128 days, compared to 138 days for those without mutations. First-line targeted therapy was associated with the longest median survival (1128 days), whereas best supportive care was linked to the shortest survival (46 days). Overall, patients in the MP-NGS group exhibited a trend toward improved survival compared to those in the SMP group, with a median OS of 672 days versus 138 days, respectively (log-rank p=0.053). According to the Cox proportional hazards model, the presence of actionable mutations remained an independent predictor of improved survival (HR: 0.48; 95% CI: 0.25-0.96; p=0.027), whereas the molecular diagnostic method (MP-NGS vs. SMP; HR: 0.99; p=0.924) and first-line therapy (HR: 1.13 across therapy types; p=0.588) were not statistically significant. All VIF values were below 5, indicating acceptable multicollinearity. Full details are available in Supplementary Tables 1 and 2 and Supplementary Figure 1.

## DISCUSSION

The present study offers a detailed comparative analysis of two molecular profiling strategies-SMP and MP-NGS-using minimally invasive EBUS-TBNA-derived samples from patients with stage IV NSCLC. Our findings highlight the superior performance of MP-NGS in identifying actionable mutations, detecting a wider array of genetic alterations, and facilitating access to personalized therapies, which may contribute to improved clinical outcomes, including a potential survival benefit.

The clinical and epidemiological characteristics of our cohort are consistent with those reported in similar patient populations,^20^ ^,^ ^21^ supporting the representativeness of our findings. While the retrospective nature of this study limited control over participant inclusion and group allocation, the comparative analysis of clinical and epidemiological variables revealed no significant differences between the two groups (Table 1), further reinforcing the internal validity of our results.

When comparing the performance of both methodologies, our findings highlight the superiority of MP-NGS over SMP in optimizing the use of EBUS-TBNA-derived samples. MP-NGS achieved a significantly higher sample adequacy rate (100% vs. 62.2%; p=2 × 10^-5^; Table 2) and identified more actionable mutations in *EGFR* (32.8%) and *ALK* (8.2%) compared to SMP (15.5% and 6.7%, respectively) (Table 2; Figure 1). Moreover, MP-NGS detected a broader spectrum of mutations in 88.5% of patients, with 14.7% harboring more than one, underscoring its enhanced sensitivity and efficiency in identifying emerging actionable targets.^22^ ^,^ ^23^ ^,^ ^24^

In order to directly compare the two methods, this study restricted the definition of actionable mutations to *EGRF*, *ALK*, and *ROS1*, in accordance with the minimum requirements outlined in international guidelines.^6^ However, the field of targeted therapy for NSCLC continues to evolve, with new actionable mutations being identified regularly.^25^ For instance, *RET* rearrangements and *BRAF* mutations-assessed only through MP-NGS in our sample-are already targetable,^26^ ^,^ ^27^ ^,^ ^28^ as observed in our cohort (Figure 2). Additionally, MP-NGS identified *KRAS* mutations, including the G12C variant in 8.2% of patients, which are increasingly actionable with inhibitors such as sotorasib, showing promising clinical outcomes.^29^ ^,^ ^30^ Furthermore, the simultaneous mutations identified via MP-NGS in several patients (Figure 1) highlight the heterogeneity of NSCLC and open possibilities for sequentially targeting multiple pathways, reinforcing the value of this profiling method.^31^

One notable finding in our study was the progressive decline in sample adequacy throughout the sequential steps of the SMP method, with the lowest adequacy observed for *ROS1* testing (62.2%) (Table 2). This trend aligns with previous reports^11^ ^,^ ^13^ and underscores the critical challenge of sample exhaustion, which is particularly relevant when dealing with limited material such as EBUS-TBNA-derived specimens. Sample depletion often results from the hierarchical testing order, in which IHC, PD-L1 assessment, and *EGFR* analysis are prioritized, frequently leaving insufficient material for FISH-based *ALK* and *ROS1* evaluations.^32^ An indirect indicator of this limitation is the discrepancy in *ALK* mutation detection rates between MP-NGS (8.2%) and SMP (6.7%). Similar findings have been reported in other studies, particularly when *ALK* is assessed by IHC, which is prone to false negatives.^25^ ^,^ ^33^ FISH, on the other hand, is generally highly sensitive and specific, provided that samples have adequate tumor content.^25^ Although the lower detection rate observed in the SMP group may partly reflect random heterogeneity inherent to the study’s retrospective design, we hypothesize that it also stems from the intrinsic limitations of EBUS-TBNA’s sampling capacity, compounded by the issue of sample exhaustion discussed above. Notably, MP-NGS effectively overcame these challenges, achieving a sample adequacy rate of 100%.

The median TAT was 17 days for SMP and 23 days for MP-NGS. Although this difference was not statistically significant, the shorter TAT for SMP likely reflects cases in which positive *EGFR* results concluded testing early, eliminating the need for further molecular analyses (Table 2). Additionally, unlike SMP, which was performed in-house, MP-NGS was outsourced, leading to longer processing times due to shipping and external handling-an issue previously documented in the literature.^34^ When compared with international guidelines and published benchmarks,^35^ these differences become more pronounced. Most studies report median TATs for NGS of around 10 days,^36^ which is substantially shorter than the values observed in our cohort. These discrepancies highlight real-world challenges in the timely diagnosis and treatment of NSCLC, especially in institutions where advanced molecular platforms are either not fully integrated or rely on external laboratories. Addressing these limitations will require coordinated strategies to optimize molecular workflows, including wider adoption of in-house MP-NGS platforms and reflex testing protocols to accelerate result turnaround times.^37^ In parallel, the development of ultra-rapid multiplex PCR platforms represents a promising complementary approach.^38^ These emerging technologies may enable broader genomic profiling-in some cases using existing RT-PCR infrastructure^38^-with the potential to deliver clinically actionable results within a markedly reduced TAT. 

The treatment data revealed distinct patterns between the two profiling methods. The MP-NGS group received more targeted therapies (44.26% vs. 22.2%; p=0.038), suggesting that MP-NGS may facilitate more personalized treatment strategies by identifying a broader range of actionable mutations (Figure 2), which may have influenced survival outcomes. Indeed, the Kaplan-Meier analysis showed a trend toward improved survival in the MP-NGS group (median OS: 672 vs. 138 days; log-rank p=0.053). Although this difference did not remain significant in the multivariable Cox model (HR: 0.99; p=0.924), the presence of actionable mutations was independently associated with OS in both models. As previously documented,^39^ ^,^ ^40^ this finding suggests that the survival advantage associated with MP-NGS is primarily mediated by factors such as the identification of actionable mutations and improved access to targeted therapies (Supplementary Tables 1 and 2; Supplementary Figure 1), ultimately reinforcing the clinical value of this method.

This study has some limitations inherent to its retrospective and uncontrolled design. Additionally, the relatively small sample size and the evolving treatment landscape of NSCLC-particularly the growing use of targeted therapies-may have influenced the outcomes.^39^ ^,^ ^40^

In spite of these constraints, the real-world nature of this study provides valuable insights into the clinical management of advanced NSCLC. Specifically, our findings highlight the superior performance of MP-NGS over SMP in detecting actionable mutations and facilitating access to personalized treatments. Although MP-NGS was associated with a longer TAT due to external processing requirements, its broader mutation coverage and greater sensitivity underscore its clinical utility in the evolving field of personalized NSCLC therapy. Moreover, the observed trend toward improved survival in the MP-NGS group further supports the potential advantages of this method over SMP, particularly in scenarios where only limited samples are available from minimally invasive procedures such as EBUS-TBNA. 

Future research should focus on evaluating the cost-effectiveness and accessibility of MP-NGS, particularly in less specialized centers, to guide strategies for its broader and more effective implementation. Additionally, as molecular diagnostics continue to evolve, future studies should explore the comparative performance, feasibility, and clinical impact of emerging genomic technologies alongside MP-NGS.
